# Expression of multidrug resistance-associated proteins and their relation to postoperative individualized chemotherapy in gastric cancer

**DOI:** 10.1186/1477-7819-12-307

**Published:** 2014-10-11

**Authors:** Pengfei Yu, Yian Du, Xiangdong Cheng, Qiming Yu, Ling Huang, Ruizeng Dong

**Affiliations:** Department of Abdominal Surgery, Zhejiang Cancer Hospital, 38# Guangji Road, Hangzhou, 310022 China

**Keywords:** stomach neoplasms, multidrug resistance-associated proteins, chemotherapy, prognosis

## Abstract

**Background:**

Adjuvant chemotherapy could reduce residual tumor cells and prevent relapse, however, not all patients are suitable for adjuvant chemotherapy. Screening appropriate patients based on molecular markers for individualized adjuvant chemotherapy is necessary.

**Methods:**

Between June 2002 and June 2004, 119 patients who underwent radical gastrectomy were retrospectively analyzed. Some patients had adjuvant chemotherapy based on platinum and 5-FU for four to six cycles. Topoisomerase II (ToPo II) negative, multidrug resistance protein (MRP) positive and glutathione S-transferase π (GST-π) positive were regarded as three risk factors that may be associated with chemotherapy resistance and poor prognosis. Patients were divided into two groups: a high-risk group (≥2 risk factors) and a low-risk group (<2 risk factors), and tumor recurrence and patient survival time of the two groups were analyzed.

**Results:**

The average recurrence time of the low-risk group was significantly longer than that of the high-risk group (21.29 ± 11.10 versus 15.16 ± 8.05 months, *P* <0.01). The 3-year and 5-year survival rates of the high-risk group were 57.4% and 42.6%, however, it had no significant difference compared to 66.2% and 58.5% of the low-risk group (*P* >0.05). In the high-risk group, the 3-year survival rates of patients with/without chemotherapy were 62.1% and 52.0% and the 5-year survival rates were 44.8% and 40.0%, respectively, but the difference was not statistically significant (*P* >0.05). In the low-risk group, the 3-year survival rates of patients with/without chemotherapy were 81.2% and 51.5%, and the 5-year survival rates were 71.9% and 45.5%, respectively, these differences were statistically significant (*P* <0.05).

**Conclusions:**

Combined detection of the multidrug resistance (MDR)-related proteins ToPo II, MRP and GST-π may be prospectively valuable for postoperative individualized chemotherapy and in further predicting the outcomes of gastric cancer patients.

## Background

There is still a high risk of recurrence and metastasis after radical gastric surgery, and adjuvant chemotherapy may reduce postoperative residual tumor cells and prevent relapse [1, 2]. In recent years, new results of randomized controlled studies indicate that postoperative chemotherapy can improve the prognosis of the patients [3, 4]. These results have been accepted in the NCCN Gastric Cancer Clinical Practice Guidelines and are recommended as the basis of postoperative treatment programs. However, some issues still need to be addressed: (1) subgroup analysis indicates that some patients (female, node-negative, late stage, older, *etcetera*) do not benefit from adjuvant therapy and (2) problems occur with chemotherapy toxicity and compliance, which causes some patients to withdraw from treatment because of adverse events.

So, postoperative adjuvant chemotherapy is beneficial for some patients, however, it may increase the treatment burden and reduce the immunity of other patients. Therefore, it is too early to determine a program as standard adjuvant chemotherapy for gastric cancer. There are still many issues that need high-quality research to answer before individualized adjuvant chemotherapy becomes standard. What is particularly worth mentioning is that the ToGA study has confirmed the value of Herceptin in the treatment of advanced gastric cancer [5]. Recently, Deng and colleagues provided for the first time a detailed molecular map of genomic alterations in gastric cancer, which revealed several promising targets for subtype-specific therapies [6]. Screening appropriate patients based on molecular markers will become a major research direction for individualized chemotherapy [7].

Multidrug resistance-associated proteins topoisomerase II (ToPo II), multidrug resistance protein (MRP) and glutathione S-transferase π (GST-π) are the basis of multidrug resistance in malignant tumors [8, 9]. It had been confirmed that MRP and GST-π overexpression, and decreased expression of ToPo II are important mechanisms mediating multidrug resistance [10]. Therefore, we carried out this study of multidrug resistance (*MDR*) gene-associated proteins in postoperative individualized treatment for gastric cancer.

## Methods

### Patients and tissue samples

Between June 2002 and June 2004, a total of 119 patients who underwent radical gastrectomy at the Department of Abdominal Surgery, Zhejiang Cancer Hospital, were retrospectively analyzed. Of these patients, 77 cases were males and 42 cases were females, ages 25 to 78 years (mean 57.3 ± 6.7 years). Phase I/II included 39 cases and Phase III/IV included 80 cases; lesions ≥5 cm were found in 76 cases and lesions <5 cm were found in 43 cases; and patients with/without lymph nodes metastases were identified for 93 cases and 26 cases, respectively. None of the patients received preoperative chemotherapy or other treatment for the tumor, and some patients had adjuvant chemotherapy based on platinum and 5-flurouracil (5-FU) for four to six cycles. Written informed consent was obtained from all the study participants. The study was approved by the Ethics Committee of Zhe Jiang Cancer Hospital.

### Immunohistochemical staining

The antibodies used in this study were purchased from GBI Company (Golden Bridge International, Inc., Mukilteo, WA, USA). Immunohistochemical staining was carried out on the formalin-fixed, 4-μm-thick, paraffin-embedded tissue specimens. Pancreas, colon, and ovary samples were used as positive controls for Topo II, MRP, and GST-π, respectively. The specimens were evaluated independently by two pathologists in a blind fashion. Only cells with brown-colored staining were considered as positive. The intensity of expression of *MDR*-related proteins was stratified into four categories that were scored as follows: 1) negative (-) had no appreciable cytomembrane, nuclear or cytoplasmic staining or had staining in <10% of neoplastic cells; 2) 1+ had appreciable staining in 10 to 25% of neoplastic cells; 3) 2+ had appreciable staining in 25 to 75% of neoplastic cells; and 4) 3+ had appreciable staining in >75% of neoplastic cells.

### Patient follow-up

Patients received routine follow-up after radical gastrectomy once every quarter for two years and thereafter, once every half year (patients who received chemotherapy were followed up with chemotherapy cycles). ToPo II negative, MRP positive and GST-π positive were regarded as three risk factors that may be associated with chemotherapy resistance and poor prognosis. Patients were divided into two groups: the high-risk group (≥2 risk factors) and the low-risk group (<2 risk factors), and the tumor recurrence and patient survival time of the two groups were analyzed.

### Statistical analysis

All the experiment data were integrated into a comprehensive data set. Numerical data were recorded directly and measurement data were described as median and range. Statistical analysis was performed on SPSS software version 16.0 (SPSS Inc. Chicago, IL), and *P* <0.05 was considered as statistically significant.

## Results

**Figure 1:**
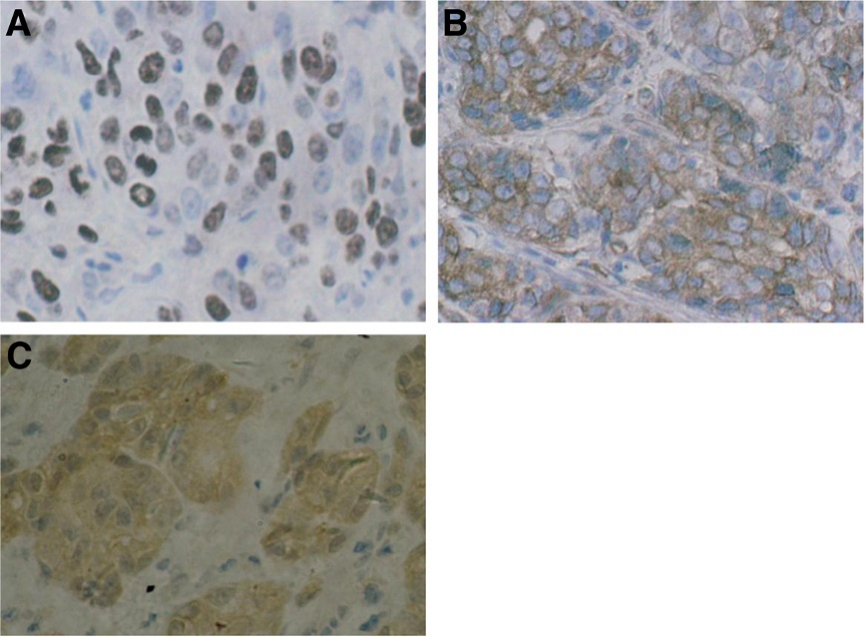
**Immunohistochemical staining of multidrug resistance-associated proteins. A** Immunohistochemical staining of ToPo II was identified in the cell nucleus (original magnification × 400). **B** Immunohistochemical staining of MRP was recognized to be expressed in the cytoplasm of malignant cells (original magnification × 400). **C** Immunohistochemical staining of GST-π was recognized to be expressed in the cytoplasm of malignant cells (original magnification × 400).

The positive staining of ToPo II was recognized to be expressed in the cell nucleus (Figure 1A), whereas MRP and GST-π were expressed in the cytoplasm of malignant cells (Figure 1B and Figure 1C).

The expression rate of ToPo II in normal tissues (75.1%) was higher than that in tumor tissues (73.9%) (not significant, *P* >0.05). When comparing the well-, moderately and poorly differentiated degree of expression, a significant correlation was shown between ToPo II expression and the level of differentiation (86.3%, 64.5% and 64.9%, respectively, *P* <0.05). As for MRP, the positive rate was 42.9% in tumor tissues whereas all the normal gastric tissues were not stained. No significant differences in MRP expression were found in relation to the clinicopathological factors. The positive rate of GST-π in patients with gastric cancer was higher than that of the normal tissues (51.3% versus 23.2%, *P* <0.05). Significant differences in GST-π expression were also found in relation to sex (male versus female, 59.7% versus 35.7%, *P* <0.05) and differentiation (well, moderately and poorly, 40.5%, 41.9%, and 64.7%, respectively, *P* <0.05) (Table 1).

**Table 1:** **The expression of ToPo II,MRP, and GST-π and their relationship with clinicopathological factors**

Clinicopathologic Features	Cases	ToPo II			MRP			GST-π		
		+(%)	χ2	***P***	+(%)	χ2	P	+(%)	χ2	***P***
Sex										
Male	77	54(70.1%)			29(37.7%)			46(59.7%)		
Female	42	34(81.0%)	1.65	0.198	22(52.4%)	2.40	0.121	15(35.7%)	6.27	0.012*
Age										
≤50 y	33	24(72.7%)			16(48.5%)			14(42.4%)		
>50 y	86	64(74.4%)	0.03	0.851	35(40.7%)	0.59	0.442	47(54.7%)	1.42	0.232
Tumor size										
≥5 cm	76	59(77.6%)			35(46.1%)			40(52.6%)		
<5 cm	43	29(67.4%)	1.48	0.224	16(37.2%)	0.88	0.349	21(48.8%)	0.16	0.691
Differentiation										
Well	51	44(86.3%)			20(39.2%)			33(40.5%)		
Moderately	31	20(64.5%)			13(41.9%)			13(41.9%)		
Poorly	37	24(64.9%)	7.04	0.029*	18(48.6%)	0.79	0.672	15(64.7%)	6.47	0.039*
TNM staging										
I/II	39	25(64.1%)			17 (43.6%)			16(41.0%)		
III/IV	80	63(78.8%)	2.92	0.087	34(42.5%)	2.57	0.109	45(56.3%)	2.43	0.119
Lymph node										
Positive	93	71(76.3%)			40(43.0%)			47(50.5%)		
Negative	26	17(65.4%)	1.27	0.260	11(42.3%)	0.004	0.949	14(53.8%)	0.09	0.765

*P < 0.05.

The 3- and 5-year survival rates of the 119 patients were 57.3% and 49.2%, respectively. No statistical difference was observed between single protein (ToPo II、MRP or GST-π) expression and the recurrence or survival time. When patients were divided into two groups: the high-risk group (≥2 risk factors) and the low-risk group (<2 risk factors), the average recurrence time of the low-risk group was 21.29 ± 11.10 months and was significantly longer than 15.16 ± 8.05 months of the high-risk group (*P* <0.01). The 3-year and 5-year survival rate of the high-risk group was 57.4% and 42.6%; however, there was no significant difference compared to the 66.2% and 58.5%of the low-risk group (*P* >0.05).

**Figure 2:**
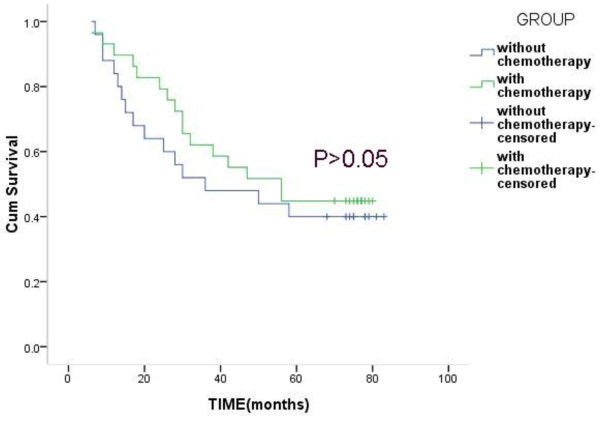
**Overall survival of patients with or without chemotherapy in the high-risk group.**

**Figure 3:**
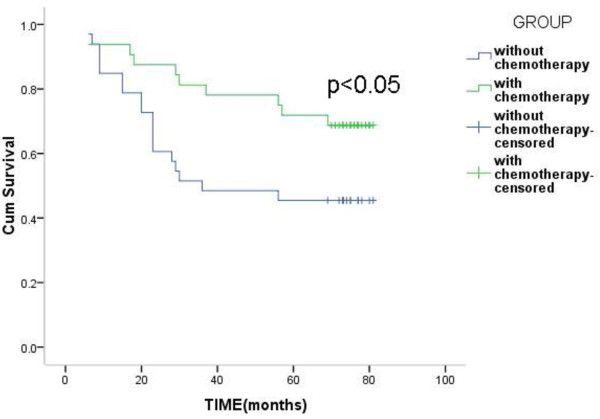
**Overall Survival of patients with or without chemotherapy in the low-risk group.**

In the high-risk group, the 3-year survival rates of patients with chemotherapy and patients without chemotherapy were 62.1% and 52.0%, and the 5-year survival rates were 44.8% and 40.0%, but the difference was not statistically significant (*P* >0.05) (Figure 2). In the low-risk group, the 3-year survival rates of patients with chemotherapy and patients without chemotherapy were 81.2% and 51.5%, the 5-year survival rates were 71.9% and 45.5%, and the difference was statistically significant (*P* <0.05) (Figure 3).

## Discussion

Adjuvant chemotherapy after operation has been considered as necessary to eliminate systemic micrometastases and remnant malignant cells to the fullest extent possible, ultimately improving survival [11, 12]. Unfortunately, so far, this kind of adjuvant treatment strategy has been disappointing as a result of multidrug resistance (MDR) of malignant cells to different chemotherapeutic agents [13, 14]. Therefore, detection and evaluation of *MDR* genes or proteins may help guide adjuvant chemotherapy in gastric cancer and determine the prognosis of patients.

MRP, one of the most studied mechanisms of MDR, acts as an ATP-dependent outward transport pump and decreases intracellular accumulation of drugs by reducing the co-transport mechanism of glutathione [15]. Several previous studies have also indicated that overexpression of MRP most frequently predicts MDR. MRP confers resistance to alkylating agents, cyclophosphamide and other drugs [16]. GST-π is a multifunctional enzyme that plays a critical role in cellular detoxification by catalyzing the conjugation of reduced glutathione to hydrophobic and electrophilic compounds [17]. GST-π is considered to be associated with the efflux of cis-diaminodichloroplatin (CDDP), flurouracil and doxorubicin (DOX) through ATP-binding cassette transporters [18]. ToPo II is the target of several anticancer agents, such as doxorubicin, VM26, VP16 and mitoxantrone [19]. The decreased expression of ToPo II and changes in enzyme activity result in the dissociation of cleavable complexes and reduced DNA damage, and finally cause the drug resistance [20].

In our study, statistical analysis indicates that none of the three proteins were significantly correlated with the recurrence and survival rates, so the determination of a single indicator of the effectiveness of adjuvant chemotherapy is difficult. Because ToPo II negative, MRP positive and GST-π positive were regarded as three risk factors that may be associated with chemotherapy resistance and poor prognosis, these patients were divided into two groups: the high-risk group (≥2 risk factors) and the low-risk group (<2 risk factors). The recurrence time of the low-risk group was significantly longer than that of the high-risk group, suggesting that the decreased expression of ToPo II and high expression of MRP and GST-π was associated with tumor invasion, recurrence and poor prognosis, and this conclusion had been confirmed in ovarian cancer [21]. In the low-risk group, the 3-year and 5-year survival rate of patients with chemotherapy was higher than that of the patients without chemotherapy. This result indicated that 5-Fu and platinum-based postoperative chemotherapy can increase survival benefits for patients in the low-risk group. Chemotherapy resistance was rare in these patients, and in theory, postoperative chemotherapy can be done fully fit, and the prognosis of the patient will be significantly improved. In the high-risk group, the 3-year and 5-year survival rate of patients with chemotherapy was higher than that of the patients without chemotherapy, but the difference was not statistically significant. Therefore, the 5-Fu and platinum-based adjuvant chemotherapy did not improve the prognosis of the high-risk group, and for such patients, postoperative chemotherapy needs to be carefully discussed and selected. These conclusions were based on a small number of cases and may have some limitations. A large sample of patients is being followed up in our center, and detailed results, including the subgroup analysis (gender, lymph node metastasis, staging, *etcetera*.), will be reported in the near future.

## Conclusions

Therefore, combined determination of *MDR*-related proteins ToPo II, MRP and GST-π may be prospectively valuable for optimizing chemotherapy regimes and further predicting the outcomes of patients. Further research should focus on the combined detection of molecular markers (such as HER-2, *MDR*-related proteins, RTK/RAS signaling molecules, *etcetera*.) for individualized chemotherapy and carry out multicenter clinical trials,the results may be exciting.

## Consent

Written informed consent was obtained from the patient for the publication of this report and any accompanying images.

## Abbreviations

CDDP: CIS-diaminodichloroplatin
DOX: doxorubicin
GST-π: glutathione S-transferase π
MDR: multidrug resistance
MRP: multidrug resistance protein
ToPo II: topoisomerase II
5-FU: 5-flurouracil.
